# Electrochemical Behavior of Janus Kinase Inhibitor Ruxolitinib at a Taurine-Electropolymerized Carbon Paste Electrode: Insights into Sensing Mechanisms

**DOI:** 10.1021/acsabm.4c00186

**Published:** 2024-04-06

**Authors:** Hasret Subak, Pınar Talay Pınar

**Affiliations:** Department of Analytical Chemistry, Faculty of Pharmacy, Van Yuzuncü Yil University, Zeve Campus, 65080 Van, Turkey

**Keywords:** Janus kinase inhibitor, ruxolitinib, electrochemical oxidation, taurine, electropolymerization

## Abstract

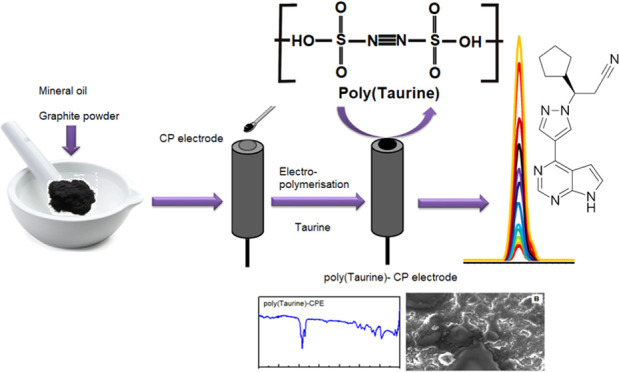

Ruxolitinib (RXL) is a Janus kinase inhibitor used for treating intermediate- or high-risk myelofibrosis. This study presents an electrode modified with electrochemically polymerized taurine on a carbon paste electrode via cyclic voltammetry (CV). The surface characterization of the poly(taurine)-CP electrode was evaluated by using electrochemical (electrochemical impedance spectroscopy—EIS, CV), morphological (scanning electron microscope—SEM), and spectroscopic (Fourier-transform infrared spectroscopy—FT-IR) techniques. Under optimized conditions, RXL exhibited good linearity within the 0.01–1.0 μM concentration range, with a limit of detection (LOD) of 0.005 μM. The proposed electrochemical sensor demonstrated excellent selectivity, accuracy, precision, and repeatability. Furthermore, it effectively detected RXL in human urine and pharmaceutical samples.

## 1 Introduction

The cytoplasmic protein kinase family comprises four Janus kinase (JAK) proteins. These proteins play a crucial role in cell signaling, particularly in the activation of signal transducers and activators of transcription (STAT) proteins. Upon activation by JAK proteins, STAT proteins translocate to the cell nucleus, where they stimulate the transcription of target genes. Ruxolitinib (RXL; Figure S1) is a potent and selective inhibitor of both JAK1 and JAK2. Its primary mode of action involves the inhibition of the JAK-mediated phosphorylation of STAT proteins. Consequently, ruxolitinib disrupts cell division and promotes apoptosis, thereby exerting its therapeutic effects.^1−4^ RXL received its first approval from the US Food and Drug Administration (FDA) in 2011 for the treatment of myelofibrosis. Subsequent approvals followed in 2014 for polycythemia vera and in 2019 for steroid-refractory graft-versus-host disease. Currently, numerous novel therapeutic applications of RXL are under investigation, particularly in diseases where its immune-modulating effects may offer significant benefits. These include conditions such as COVID-19 and dermatological autoimmune diseases.^5−7^

Amino acids are fundamental building blocks of peptides and are essential components for all organisms. Their structures contain various functional groups such as −COOH, −OH, –NH2, and −CH*x*, allowing for polymerization. Taurine (2-aminoethanesulfonic acid), a derivative of cysteine, features a thiol group. Apart from its crucial role in the central nervous system, taurine is indispensable for cardiovascular, skeletal muscle, and retinal functions. Additionally, it serves as a food nutritional enhancer and exhibits pharmacological properties, making it a common drug.^8^ Taurine, a β-amino acid found abundantly in human and animal tissues, serves as an efficient green bio-organic catalyst for producing certain biologically active acid derivatives.^9^ Taurine is favored by many researchers for its electropolymerization on the electrode surface, which is attributed to the presence of its aminic and –SO3– groups in its structure. In recent years, polymer film-modified electrodes have gained recognition for their diverse applications in the field of electrochemical sensors. These electrodes have demonstrated the ability to significantly enhance the electrocatalytic properties of analytes, expedite reaction rates, and bolster the stability of electrode response.^10^ Moreover, the cost-effectiveness of these sensors remains low due to the straightforward electrodeposition procedures involved.^11^ Additionally, the electropolymerization of specific organic molecules holds promise for the development of novel biosensor electrodes. Consequently, the thickness, permeability, and charge transport characteristics of modified polymeric films can be effectively delineated. In the literature search, there are a few reports on polytaurine-modified electrode surfaces.^12−20^

In the literature survey, various analytical methods have been developed for the quantitative analysis of RXL, including high-performance liquid chromatography (HPLC), liquid chromatography–mass spectroscopy (LC–MS), fluorescence techniques, and microwell-based spectrofluorimetric methods.^21−27^ While these methods are effective and sensitive, they often involve high costs and are not environmentally friendly due to the extensive use of organic solvents. Additionally, they require long analysis times and expertise in handling analytical devices.

In contrast, electrochemical methods offer a more environmentally friendly approach and are preferred for analyzing electroactive compounds in various fields, such as agriculture, food, pharmaceuticals, and healthcare.^28−32^ Among electroanalytical techniques, voltammetry stands out as one of the most commonly used methods, particularly in drug analysis. These electrochemical methods offer several advantages, including shorter analysis times, lower costs, reduced use of organic solvents, high sensitivity, precise analytical capabilities, and the ability to analyze small sample volumes.^33−36^

Carbon paste electrodes (CPEs) are highly versatile materials extensively utilized in electrochemical applications.^37,38^ Modifying the surfaces of these electrodes can significantly enhance their sensitivity, surface area, conductivity, and overall analytical performance.^39−41^ One common method involves coating the electrode surface with polymers, which serves to bolster stability and impart selectivity to the electrode. The electrode surface can undergo polymer coating to enhance stability and confer selectivity.^42−44^

In this study, we utilized poly(taurine) as a novel conductive polymer film for the electro-oxidation and determination of RXL. The proposed sensor was applied to standard spiked urine and pharmaceutical samples to study the electrochemical behaviors of RXL with high sensitivity, selectivity, rapidity, reliability, and reproducibility.

## 2 Materials and Methods

### 2.1 Reagents and Apparatus

The RXL standard pharmaceutical active ingredient was purchased from ChemScene (Türkiye, Cat. No: CS-0864; purity: 99.99%). Taurine (purity ≥99.0%, Merck, Türkiye) was used in the modification of the working electrode, and methanol (purity ≥99.8%, Merck, Türkiye) was used to prepare the standard solution. 0.1 M acetate buffer (ABS, pH 3.7, 4.7, 5.7), 0.1 M phosphate buffer (PBS pH 2.5, 7.4, 12.0), 0.1 M Britton–Robinson buffer (BRT, pH 2.0–12.0), and H_2_SO_4_ (purity: 96%, Merck) (0.1, 0.2, and 0.5 M) were used as supporting electrolytes. Additionally, dopamine (purity ≥99.0%), ascorbic acid (purity ≥99.0%), uric acid (purity ≥99.0%), lactose (purity ≥99.0%), glucose (purity ≥99.0%), potassium chloride (purity ≥99.99%), magnesium chloride (purity ≥98%), sodium sulfate (purity ≥99.0%), and potassium nitrate (purity ≥99.0%) were obtained from Sigma-Aldrich (Türkiye) to perform interference studies. The chemicals H_3_PO_4_ (85%), NaH_2_PO_4_·H_2_O (98%), CH_3_COOH (100%), HCl (37%), H_3_BO_3_ (99.5%), and Na_2_HPO_4_ (98%) used in the preparation of supporting electrolyte solutions were obtained from Sigma-Aldrich, Türkiye. Additionally, potassium ferrocyanide (99%) (K_4_[Fe(CN)_6_]·3H_2_O), potassium ferricyanide (99%) (K_3_[Fe(CN)_6_]), and potassium chloride (KCl) were used as redox probes for electrochemical characterization experiments, all purchased from Sigma-Aldrich, Türkiye.

Electrochemical studies were carried out with cyclic voltammetry (CV), square-wave voltammetry (SWV), and electrochemical impedance spectroscopy (EIS) techniques using an Autolab PGSTAT 101 (Eco Chemie, Metrohm Autolab B.V., Netherlands) electrochemical analyzer with NOVA 2.1.6 electrochemical software. The high-resolution surface images of the samples were obtained using a field emission scanning electron microscope (Zeiss VP Sigma 300 FESEM). The Fourier-transform infrared spectroscopy (FT-IR) spectrum was recorded in the wavenumbers range of 4000–500 cm^–1^ by Fourier-transform infrared spectroscopy (ATR-FTIR 8000 series, Shimadzu, Japan). The surface morphology of the modified electrodes was evaluated by scanning electron microscopy (SEM) using a LEO 438 VP (LEO Instruments, U.K.) SEM in a high vacuum mode at 20 keV.

EIS measurements were conducted across a broad frequency spectrum, ranging from 0.01 to 10,000 Hz. A potential of +0.25 V was applied, with an amplitude of 10.0 mV, to a solution containing 0.5 M KCl and 2.0 mM [Fe(CN)_6_]^(3–/4−)^ redox probe.

Electrochemical studies were carried out using a triple electrochemical cell system containing a working, counter, and reference electrode. The carbon paste electrode was used as the working electrode (BASi, MF-2010); the platinum wire (MW-1032) obtained from BASi was used as the counter electrode; and the Ag/AgCl electrode (3 M KCl; BASi, MF 2056) was used as the reference electrode. In addition, solid chemical substances were weighed with a Vibra brand electronic scale with a sensitivity of 0.01 mg. ISOLAB model ultrasonic bath was used to clean the working electrode and dissolve some substances. A WTW inoLab pH7110 digital pH meter was used to adjust the pH of the solutions.

**Scheme 1:**
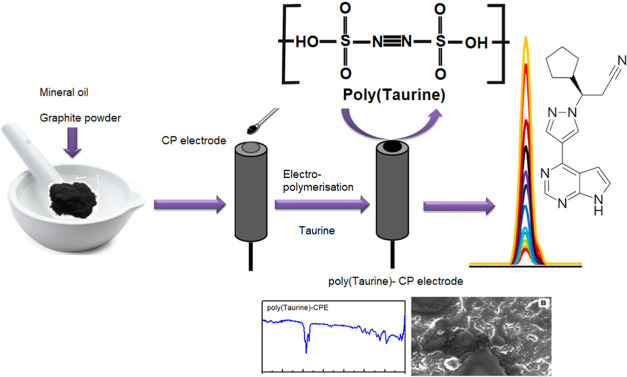
Illustrates the Process used to Fabricate the Poly(taurine)-CP Electrode

Carbon paste was prepared by homogeneously mixing 70% (w/w) graphite powder and 30% (w/w) mineral oil and pressed into the electrode.^45^ The electrode surface was turned into a homogeneous surface with a wax paper. The surface of the electrode was renewed before each experiment. For electrode modification, the carbon paste electrode selected as the working electrode was placed in 0.1 M pH 7.4 phosphate buffer solution containing taurine, a modifier, at a 1 mM concentration, and electrode connections were made. With the cyclic voltammetry technique used for the modification process, voltammograms were taken for 5 cycles under a nitrogen atmosphere, in the voltage range of −1.5– to 2.0 V, at a scan rate of 100 mV s^–1^. After the electropolymerization process, the poly(taurine)-modified carbon paste electrode was washed with water to remove residues that may have come from the monomer solution (Scheme 1).

### 2.2 Preparation of Real Samples for Voltammetric Analysis

#### 2.2.1 Human Urine

The sample was taken from healthy (drug-free urine samples) laboratory volunteers and was preserved in the refrigerator. For protein precipitation, acetonitrile was added to the samples, and centrifugation was performed at 8000 rpm for 5 min. 0.5 mL of the supernatant was withdrawn and then diluted to a total volume of 25 mL in a volumetric flask containing a pH 4.7 ABS solution. The urine sample underwent a simple dilution with the supporting electrolytes (1:100). Urine samples spiked with an appropriate amount of standard RXL solution were analyzed by using the SWV technique with the proposed method, eliminating the requirement for any additional pretreatment or extraction steps.

#### 2.2.2 Tablet Sample

Three RXL tablets (JAKAVI, Novartis, Turkiye), each containing 10 mg of RXL, were weighed and ground thoroughly. The tablet powder equivalent to prepare a 1 × 10^–3^ M RXL solution was weighed and transferred to a 25.0 mL volumetric flask, then dissolved in methanol in an ultrasonic bath for 10 min. To verify the accuracy of the procedure, recovery tests were conducted by adding a known amount of the pure drug substance to the RXL tablet solution.

## 3 Results and Discussion

### 3.1 Electropolimarization of the Poly(taurine)-CP Electrode

**Figure 1:**
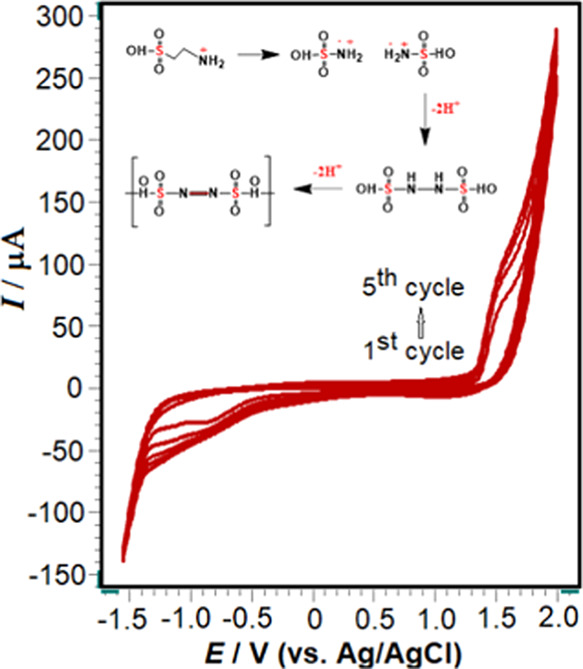
CV for the electropolymerization of poly(taurine) was conducted in a solution containing 1.0 mM monomer and 0.1 M PBS (pH 7.4) at a scan rate of 100 mV/s. The inset of the figure illustrates the possible electropolymerization reaction mechanism of poly(taurine).

The electropolymerization of taurine was conducted via cyclic voltammetry within the potential range of −1.5 to 2.0 V. During the polymerization process, a distinct oxidation peak at 1.5 V and a reduction peak at −0.84 V were observed. As the cycles progressed, these peaks contributed to the formation of a film layer on the surface of the CP electrode. The reduction peak indicates the reduction of the monomer, while the oxidation peak signifies the advancement of the polymerization reaction. After 5 cycles, the electropolymerization of taurine on the CP electrode surface reached its maximum extent. Subsequently, the resulting poly(taurine)-modified CP electrode was meticulously dried and prepared for utilization in subsequent analyses. Figure 1 displays the cyclic voltammograms of poly(taurine) and depicts the potential electropolymerization reaction mechanism as proposed by Madhu et al.^13^

### 3.2 Characterization of the Poly(taurine)-CP Electrode

**Figure 2:**
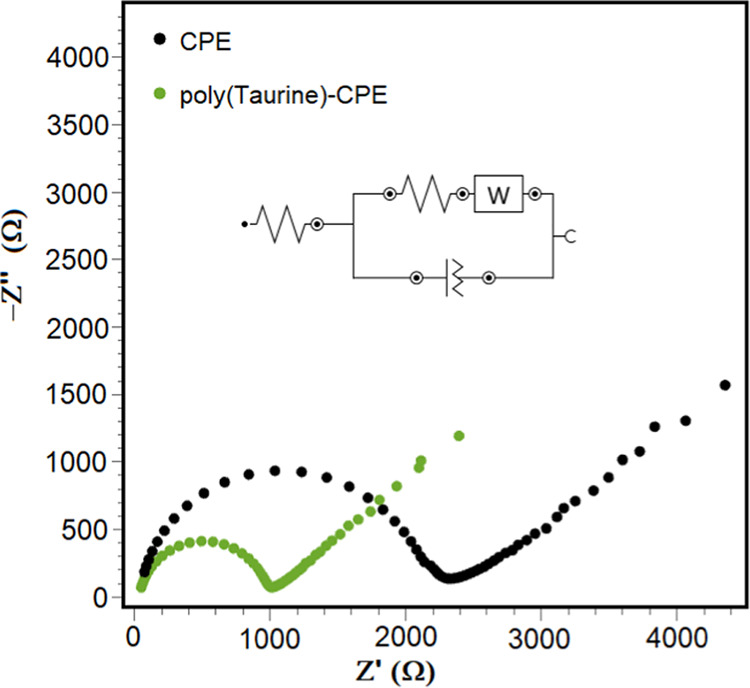
Typical Nyquist plots of the CP electrode (black) and poly(taurine)-CP electrode (green) are depicted in 2.0 mM [Fe(CN)_6_]^3–/4–^ solution containing 0.5 M KCl. The frequency range spanned from 10,000 to 0.01 Hz at a potential of 0.25 V. Inset, the Randles circuit model is illustrated, including *R*_s_, which represents the electrolyte resistance, *R*_ct_ denoting the charge transfer resistance, CPE representing the constant phase element, and *Z*_w_ representing the Warburg impedance.

EIS and CV techniques are commonly employed for the characterization of the electrode surface morphology. In the EIS and CV studies utilized for surface characterization of CP and poly(taurine)-CP electrodes, [Fe(CN)_6_]^3–/4–^ served as the redox probe. EIS stands as a robust method for investigating material properties, electrode surface reactions, and electron transfer properties, at the electrode–electrolyte interface. Nyquist plots typically exhibit two regions along the axis: the first region portrays a semicircular shape, while the second region manifests as a straight line. The semicircular segment observed at high frequencies corresponds to the process governed by electron transfer, whereas the linear portion observed at low frequencies represents the process controlled by diffusion.^46−48^Figure 2 shows the EIS spectra of both bare CP and poly(taurine)-CP electrodes in the frequency range of 0.01 to 10,000 Hz in 0.5 M KCl/2.0 mM [Fe(CN)_6_]^3–/4–^ redox probe solution. It was observed that the largest semicircle diameter was obtained on the CP electrode. The *R*_ct_ (the charge transfer resistance) value is derived from the diameter of the semicircle, and the solid line of the graphs represents the *R*_s_ (the electrolyte resistance) value. *R*_ct_ values of bare CP and poly(taurine)-CP electrodes were obtained at 2144.9 and 964 Ω, respectively. As the surface area increases as a result of the electropolymerization of taurine onto the electrode surface, the number of active sites on the electrode surface increases. This may contribute to more efficient electrochemical reactions at the electrode surface and, thus, to a reduction of charge transfer resistance. At the same time, when integrated into the electrode surface due to their high electrical conductivity, they facilitate electron transfer, allowing electrons to reach the electrode surface more easily and faster. This can contribute to faster load transfer and therefore reduced resistance. This indicates that the interfacial electron transfer rate between the poly(taurine)-CP electrode surface and the analyte increases, thereby increasing the electrocatalytic effect.

**Figure 3:**
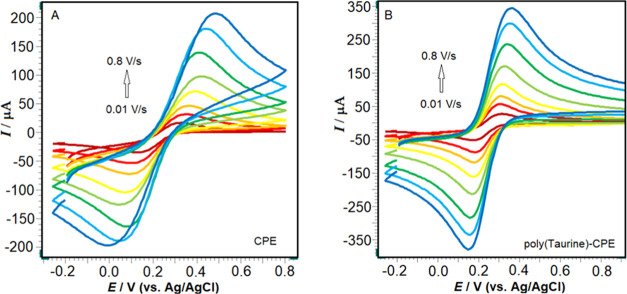
CVs of 2.0 mM [Fe(CN)_6_]^3–/4–^ in 0.5 M KCl obtained at (A) CPE, and (B) poly(taurine)/CPE at different scan rates (0.01–0.8 V/s).

Figure 3A,B illustrates the cyclic voltammograms of bare CP and poly(taurine)-CP electrodes obtained with varying scan rates in a 0.5 M KCl solution containing a 2.0 mM [Fe(CN)_6_]^3–/4–^ redox probe. Both bare CP and poly(taurine)-CP electrodes exhibited well-defined reversible redox peaks. The electroactive surface area of each electrode was determined using the CV technique at different scan rates (ranging from 0.01 to 0.8 V/s) and calculated utilizing the “Randles–Sevcik” equation (eq 1) provided below.
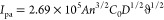
1In the equation, *I*_p_ represents the peak current, *n* is the number of transferred electrons (Fe(CN)_6_^3–/4–^ where *n* = 1), *A* is the effective surface area, *D* is the diffusion coefficient (7.7 × 10^–6^ cm^2^/s), *v* is the scan rate (V/s), and *C* is the concentration of the redox probe. Consequently, the electroactive real surface areas of CP and poly(taurine)-CP electrodes were calculated as 0.048 and 0.098 cm^2^, respectively. Linearity graphs between √*v* and *I*_pa_/*I*_pc_, as well as cyclic voltammograms for both CP and poly(taurine)-CP electrodes at a scan rate of 100 mV/s, are provided in Figures S2 and S3.

Heterogeneous electron transfer rate constants (k_et) were computed using the following formula (eq 2) utilizing data acquired from EIS
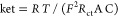
2In the formula, *R* denotes the universal gas constant (8.314 J/K/mol), *F* represents the Faraday constant (96,485 C/mol), *R*_ct_ stands for the charge transfer resistance (Ω), A signifies the electrode surface area (cm^2^), and *C* represents the concentration of [Fe(CN)_6_]^3–/4–^ (mol/cm^3^). The calculated ket values for CP and poly(taurine)-CP electrodes were determined as 1.13 × 10^–6^ and 1.34 × 10^–6^ cm/s, respectively. Consequently, it can be inferred that the ket value measured by the poly(taurine)-CP electrode surpasses that of the CP electrode, indicating that electrons exhibit higher speed in the modified electrode.

Additionally, to assess the electrocatalytic activity of the developed electrochemical sensor, the standard exchange current density (*I*_0_) can be computed using the following equation (eq 3).
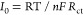
3The developed electrochemical sensor, the poly(taurine)-CP electrode, exhibits a standard exchange current density (*I*_0_) of 0.244 μA/cm^2^, which surpasses that of the bare CP electrode (0.110 μA/cm^2^), indicating significantly enhanced electrocatalytic activity of the poly(taurine)-CP electrode.^49^

**Figure 4:**
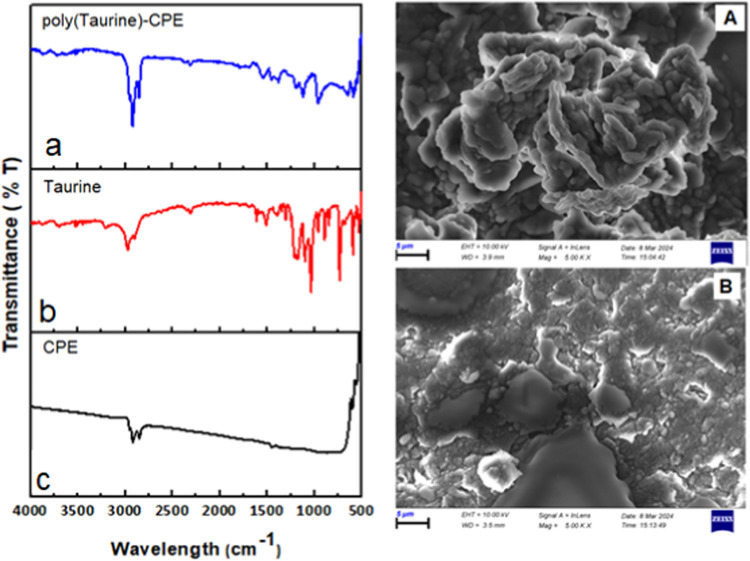
FT-IR spectra of (a) poly(taurine), (b) taurine, and (c) the CP electrode; (A) SEM images of the CP electrode surface; (B) SEM images of the polymer coating on the electrode surface.

Information regarding the chemical composition of taurine, CPE, and poly(taurine)-CPE was obtained using FT-IR. In the FT-IR spectrum provided in Figure 4b for taurine, the signals observed at 3210, 2974–2908, 1617, 1100, and 1042 cm^–1^ are assumed to originate from asymmetric N–H stretching, aliphatic C–H stretching, –NH_2_ bending, C–N bending, and −SO_3_ symmetric stretching vibrations, respectively. In the FT-IR spectrum of the poly(taurine)-CP electrode (Figure 4c), stretching and bending vibrations are observed in approximately the same regions as those of taurine. This observation suggests that the chemical structure of the taurine molecule is preserved during the coating of the electrode surface with taurine in a polymeric form.^50,51^ The surface morphologies of the unmodified and modified CP electrodes were examined by scanning electron microscopy (SEM). SEM images showed significant differences between the surface morphologies of the CPE and poly(taurine)-CPE. The CPE is dominated by a homogeneous and evenly distributed surface with fine pores (Figure 4A). After the modification, sharp lines of the polymeric structures were formed due to the electropolymerization of taurine (Figure 4B).

### 3.3 Electrochemical Behavior of RXL

**Figure 5:**
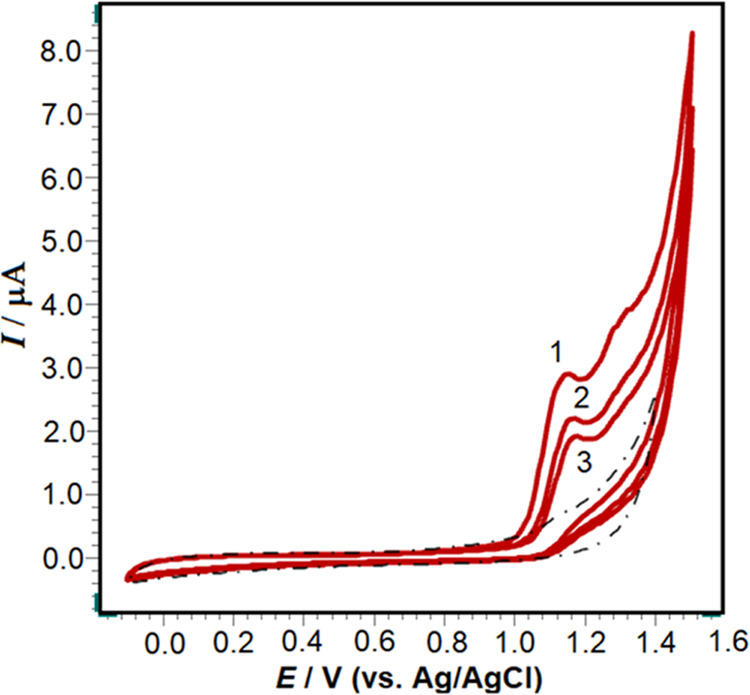
CVs of 5 × 10^–5^ M RXL at the poly(taurine)-CP electrode with three repetitions in pH 4.7 of ABS solution. The scan rate is 100 mV/s.

First, the CV technique used to investigate the electrochemical response of 5 × 10^–5^ M RXL on the poly(taurine)/CP electrode in pH 4.7 of 0.1 M ABS (the best medium for RXL analysis has been selected and will be mentioned later) is presented in Figure 5. As seen in the voltammogram, oxidation peaks of RXL were obtained at 1.12 μA at 1.15 V with the poly(taurine)/CP electrode in pH 4.7 of 0.1 M ABS and no reduction peak was observed in the cathodic scan. This result shows that the oxidation of the RXL molecule under all of the applied conditions is completely irreversible.

**Figure 6:**
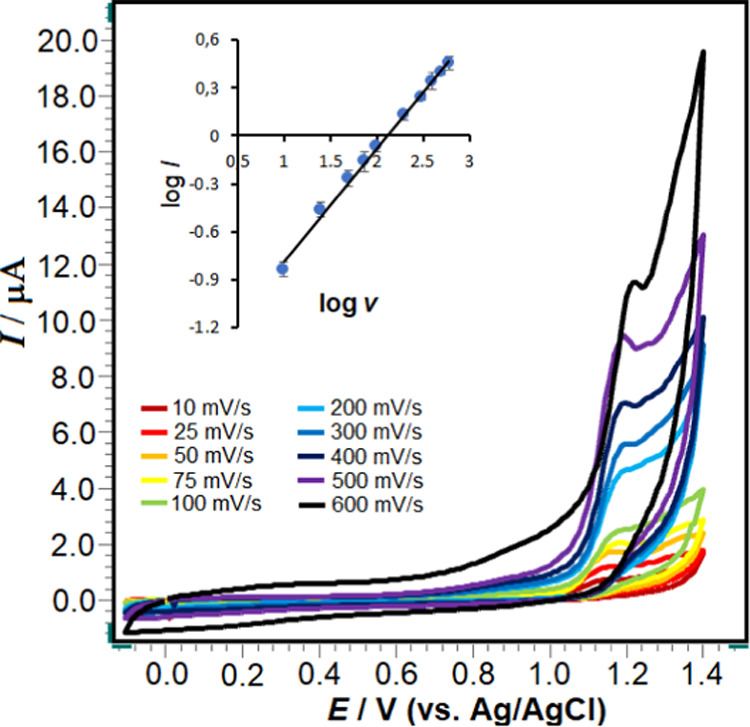
Cyclic voltammograms of 5 × 10^–5^ M RXL recorded using the poly(taurine)/CP electrode in the scan rate range of 10–400 mV/s in pH 4.7 of ABS solution. Inset: linearity graphs of log* v*–log *I*_p_.

Valuable information regarding the electrode reaction mechanism (rate determination step) can be obtained from the relationship between the peak current and scan rate. The effect of scan rate on the electrochemical oxidation of 5 × 10^–5^ M RXL was studied using different scan rates (10–600 mV/s; *n*:10) with the poly(taurine)-CP electrode in pH 4.7 of ABS solution, and the corresponding voltammograms are shown in Figure 6. √*v* – *I*_p_ (eq 4) and log *v*–log *I*_p_ (eq 5) graphs were drawn using the findings obtained from these voltammograms, and the linearity equations of these graphs are presented below.
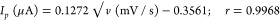
4
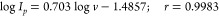
5

The linearity obtained from the *I*_p_/√*v* relationship shows that the electrochemical oxidation of the RXL molecule is diffusion-controlled under the experimental conditions studied. When the logarithmic equations of current and scan rate are examined, the slope obtained is 0.7, which shows that this reaction is not completely diffusion- or adsorption-controlled and that there is also an adsorptive effect in addition to diffusion. On the other hand, as seen from the voltammograms (Figure 6), as the scan rate increased, the oxidation peak of the RXL molecule shifted toward slightly more positive potential values. This phenomenon is, therefore, characteristic of irreversible electrode processes. The relationship between *E*_p_ and scan rates provides a lot of useful information, especially the reaction mechanism of RXL. The plot of *E*_p_ (V) vs log *v* (mV/s) follows the linear regression eq 6
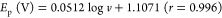
6As the process is an irreversible electrochemical reaction, the peak potential (*E*_p_) can be expressed according to Laviron’s expression (eq 7), from which the product of the number of electrons participating (*n*) and the electronic transfer coefficient (α) in the process can be calculated.
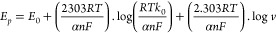
7where *T*, *R*, *F*, *E*_0_, *v*, *n*, *k*_0_, and α denote the absolute temperature, the universal gas constant, the Faraday constant, the formal redox potential, the scan rate, the number of electrons transferred, the heterogeneous transfer constant of the reaction, and the electronic transfer coefficient, respectively. By substituting the given values into the expression *E*_p_ = (2.303*RT*/α*nF*).log*v*, and considering the slope of the plot of *E*_p_ (V) vs log* v* (mV/s), the obtained α*n* value was found to be 1.06. In electrochemical irreversible reactions, α is typically equal to 0.5. Consequently, *n* = 2.12 (∼2) is determined for the oxidation of RXL.^48^

**Figure 7:**
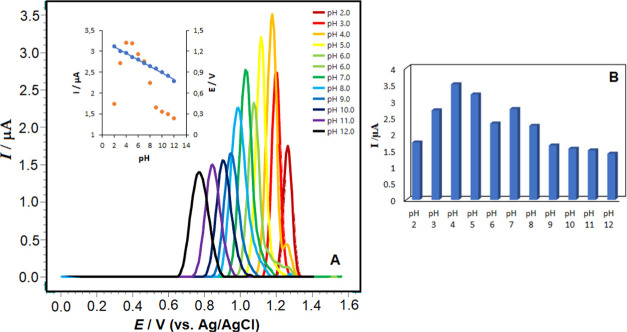
(A) Square-wave voltammograms of 1 × 10^–5^ M RXL in BR buffer (pH 2.0–12.0) and (B) histograms of 1 × 10^–5^ M RXL in BR buffer (pH 2.0–12.0). Inset: The linearity graph of RXL; Electrode, poly(taurine)-CPE; SWV parameters: 50 Hz frequency, 8 mV scan increment, and 30 mV pulse amplitude.

**Figure 8:**
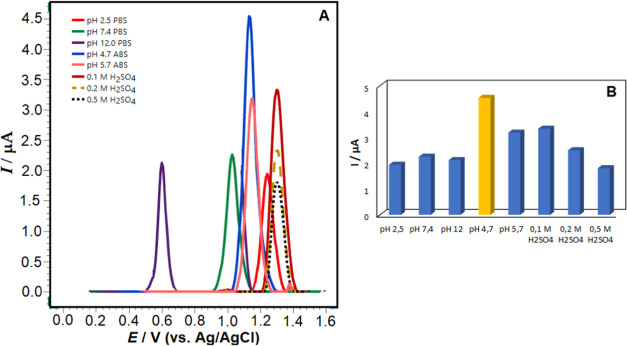
(A) Square-wave voltammograms of 1 × 10^–5^ M RXL in different pH solutions and (B) histograms of 1 × 10^–5^ M RXL in different pH solutions. Electrode, poly(taurine)-CPE; SWV parameters: 50 Hz frequency, 8 mV scan increment, and 30 mV pulse amplitude.

As is well known, the pH of the analytical solution plays a crucial role in determining whether protons participate in electrode reaction mechanisms. In studies aimed at developing a sensitive and selective voltammetric method for the determination of RXL, sharper and well-defined peaks were observed with the square-wave voltammetry (SWV) technique. The impact of pH on the anodic potential and current responses at the poly(taurine)-CP electrode was investigated in a solution containing 1 × 10^–5^ M RXL. To assess the influence of the supporting electrolyte and pH on the voltammetric behavior of RXL, SW voltammograms were recorded over the potential scan range of (0.0) to (+1.6) V for RXL solutions prepared in the appropriate supporting electrolyte. To this end, supporting electrolyte solutions of 0.1 M Britton–Robinson buffer (pH 2.0–12.0) were utilized. Figures 7A,B and 8A,B depict the effect of pH on square-wave voltammograms and histograms recorded in 1 × 10^–5^ M RXL solutions.

As seen in Figures 7 and 8A, the oxidation peak current of RXL can change with different pH values. The highest peak current was obtained in pH 4.7 ABS solution. The oxidation peak potential of RXL shifts toward more negative as pH increases. This result shows that protons on the poly(taurine)-modified carbon paste electrode surface affect the electrochemical mechanism. The effects of the supporting electrolyte on phosphate, acetate buffer, and strong acids were also investigated. Figure 8A shows the histograms of 1 × 10^–5^ M RXL in phosphate buffer (pH 2.5, 7.4, 12), acetate buffer (pH 4.7, 5.7), and 0.1, 0.2, and 0.5 M H_2_SO_4_ solutions. As can be seen from the SW voltammograms in Figures 7A and 8A, pH 4.7 ABS solution was preferred in terms of both peak morphology and peak current, and all studies were continued in this medium. When the relationship between pH and peak potential (*E*_p_) of RXL is examined, it can be seen that there is a single slope region in the pH range of 2–12 (Figure 7; inset). The linear regression equation of RXL is expressed as follows (eq 8)
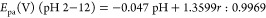
8The fact that the obtained slope (−47 mV) is close to the theoretically known 59 mV (Nerst equation) shows that the number of electrons and protons in the electrode mechanism is equal.

It is known that the p*K*_a_ value of a drug is the basic physicochemical parameter that affects many biopharmaceutical properties. p*K*_a_ affects lipophilicity, solubility, protein binding, and permeability, which directly affect pharmacokinetic properties such as absorption, excretion, distribution, and metabolism. Therefore, the p*K*_a_ value of a drug indicates the characteristic ionic form that a molecule will take at various pH values. Ruxolitinib is a 7*H*-pyrrolo[2,3-*d*]pyrimidine derivative and has an acid dissociation constant (p*K*_a_) of 5.9. Ruxolitinib is the predominant entity in humans, representing approximately 60% of the circulating drug-related material. Its excretion is 22% in feces and 74% in urine.^4,52^

**Scheme 2:**
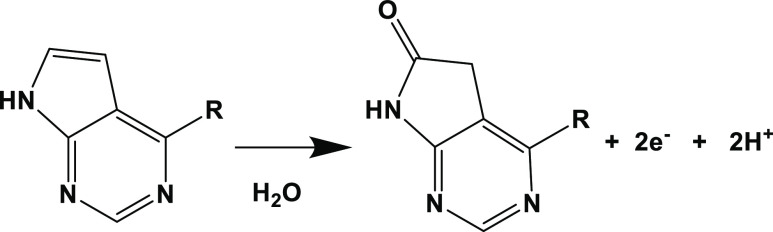
Possible Electrochemical Oxidation Reaction of RXL

In light of the above information, it can be thought that the electrochemical oxidation occurs in the pyrrolopyrimidine ring, which is the main part of the structure of RXL.^53^ Therefore, it was stated that the oxidation reaction took place as 2H^+^–2e^–^ on the poly(taurine)-CP electrode surface. The possible electrochemical oxidation reaction is illustrated in Scheme 2.

Since the signals occurring in voltammetric techniques may vary with the variables of the device used, it is necessary to optimize these variables. For this purpose, square-wave pulse variables were optimized in the range of 15–200 Hz frequency (*f*), 4–20 mV step potential (Δ*E*_s_), and 10–100 mV pulse amplitude (Δ*E*_sw_) of 1 × 10^–5^ M RXL in pH 4.7 ABS solution. The optimization process was carried out by changing one parameter at a time and keeping the others constant. By increasing the frequency, the peak current increased up to 200 Hz, but after 100 Hz, the peak current increased, but the peak morphology deteriorated. For this reason, the most appropriate frequency value was determined as 100 Hz. The best result in terms of peak shape and peak current value in the potential range of 4–30 mV was obtained at Δ*E*_s_ = 20 mV, and the best result was obtained at 40 mV in peak current in the pulse amplitude range of 10–100 mV.

### 3.4 Analytical Application of RXL

**Figure 9:**
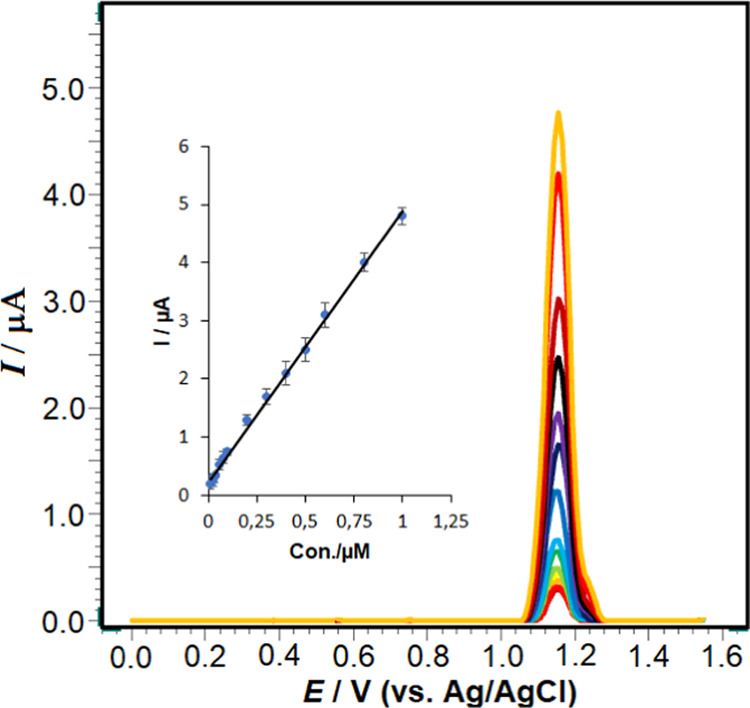
Square-wave voltammograms for RXL levels of 1.0 × 10^–8^, 2.0 × 10^–8^, 3.0 × 10^–8^, 4.0 × 10^–8^, 6.0 × 10^–8^, 8.0 × 10^–8^, 1.0 × 10^–7^, 2.0 × 10^–7^, 3.0 × 10^–7^, 4.0 × 10^–7^, 5.0 × 10^–7^, 6.0 × 10^–7^, 8.0 × 10^–7^, and 1.0 × 10^–6^ in pH 4.7 ABS. The inset shows the corresponding calibration plot for the quantitation of RXL. SWV parameters are as follows: Δ*E*_s_, 20 mV; *f*, 100 Hz; and Δ*E*_sw_, 40 mV.

To investigate the impact of RXL concentration on the oxidation peak current under optimal experimental conditions, voltammograms of RXL solutions at various concentrations were analyzed using the poly(taurine)-CP electrode in a pH 4.7 ABS solution. Calibration SW voltammograms were acquired by incrementally increasing the concentrations of RXL at +1.15 V. Figure 9 illustrates that RXL exhibits a linear relationship [*ip* (μA) = 4.6641 C (μM) + 0.2308], with a correlation coefficient (*r*) of 0.999. The linearity is observed within the range of 0.01–1.0 μM. The relative standard deviation (RSD) values for the slope and intercept of the calibration curve for RXL oxidation were determined to be 1.22 and 3.73%, respectively. These findings indicate that the poly(taurine)-CP electrode demonstrates excellent repeatability for the electrochemical oxidation of RXL. The calculated values for the limit of detection (LOD) and limit of quantification (LOQ) were determined to be 0.005 and 0.01 μM, respectively. These values were obtained by using the following equations: LOD = 3*s*/*m* and LOQ = 10*s*/*m*, where “*s*” represents the standard deviation of the peak current of the lowest concentration in the calibration curve and “*m*” is the slope of the calibration curve.

Three reports have been identified in the literature for the voltammetric detection of RXL based on electrochemical oxidation. Çorman et al. designed a molecular imprinted electrochemical sensor for the detection of RXL in 2021, and Bilge et al. designed an electrochemical sensor with different solid electrodes and Co_3_O_4_ nanoparticles in 2022.^47−49^ Comparison of the analytical performance of the developed approach with previously reported electrochemical methods for the determination of RXL is given in Table 1.

**Table 1:** Comparison of Electrochemical Methods for RXL Determination^a^

electrode	linearity range	LOD	refs
GCE	4.0–80.0 μM	5.17 × 10^–7^ M	(54)
BDDE	1.0–80.0 μM	1.92 × 10^–7^ M	(54)
GCE/MIP@PHEMA-ThyM	0.01–0.1 pM	1.91 × 10^–15^ M	(53)
SC-Co_3_O_4_-GCE	0.08–20 μM	6.73 × 10^–9^ M	(55)
poly(taurine)-CPE	0.01–1.0 μM	5.00 × 10^–9^ M	this work

a GCE: glassy carbon electrode; BDDE: boron-doped diamond electrode; GCE/MIP@PHEMA-ThyM: glassy carbon electrode/molecularly imprinted polymer@poly(2-hydroxyethyl methacrylate-*co*-thymine methacrylate); SC-Co_3_O_4_-GCE: sponges with Co_3_O_4_ nanoparticles-modified glassy carbon electrode.

This developed method demonstrates *a* significantly lower limit of detection for the target analyte (RXL) compared to existing methods, indicating higher sensitivity in detecting the analyte. Additionally, the working range of this developed method encompasses the ranges of existing methods, making it suitable for a wide range of analyte concentrations.

The repeatability, reproducibility, and stability of the poly(taurine)-CP electrode were investigated using SWV under optimized conditions (Δ*E*_s_, 20 mV; *f*, 100 Hz; and Δ*E*_sw_, 40 mV). The repeatability of the poly(taurine)-CP electrode was evaluated by conducting 10 SWV measurements of 4 × 10^–7^ M RXL. These measurements were repeated 10 times on the same day and in three different solutions on different days. Intraday and interday %RSD values of oxidation peak currents of RXL were determined as 2.12 and 3.23%, respectively. This suggests that the fabricated sensor exhibits good precision for the determination of RXL. To evaluate the reproducibility of the poly(taurine)-CP electrode, four different electrodes were prepared under the same conditions, and SWVs were recorded for 4 × 10^–7^ M RXL (Figure S4). A relative standard deviation (RSD) of 1.85% (*n* = 3) was obtained, demonstrating excellent reproducibility of the responses at the poly(taurine)-GCE. Furthermore, the long-term stability of the poly(taurine)-GCE was assessed by measuring the peak potential and current signal of 4 × 10^–7^ M RXL for three consecutive weeks (Figure S5). At the end of the third week, the peak potential for RXL oxidation remained unchanged and a relative standard deviation (RSD) of 2.02% (*n* = 3) was obtained, indicating good long-term stability of the modified electrode.

The sensitivity was determined to be 47.59 μA/μM/cm^2^ based on the ratio of slope to the active surface area, which was measured as 0.098 cm^2^ for the 1.0 cm geometric area of the poly(taurine)-CP electrode.

The surface coverage of the modified electrode refers to the amount or degree of modification that occurs on the electrode surface. Surface coverage is an important parameter that directly affects the electrochemical properties and performance of the modified electrode in various applications, such as sensing or catalysis. The total surface coverage was calculated using the following Laviron eq (eq 9).^56^
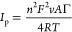
9where “*I*_p_” is the peak current (A), “*n*” is the number of electrons, “*F*” is the Faraday constant (96 485 C/mol), “*ν*” is the potential scan rate (V/s), “*A”* is the electrode area (cm^2^), “Γ” is the surface coverage (mol/cm^2^), “*R*” is the ideal gas constant (8.314 J/K/mol), and “*T*” is the temperature (K). The total surface coverage calculated was 2.23 × 10^–11^ mol/cm^2^.

**Figure 10:**
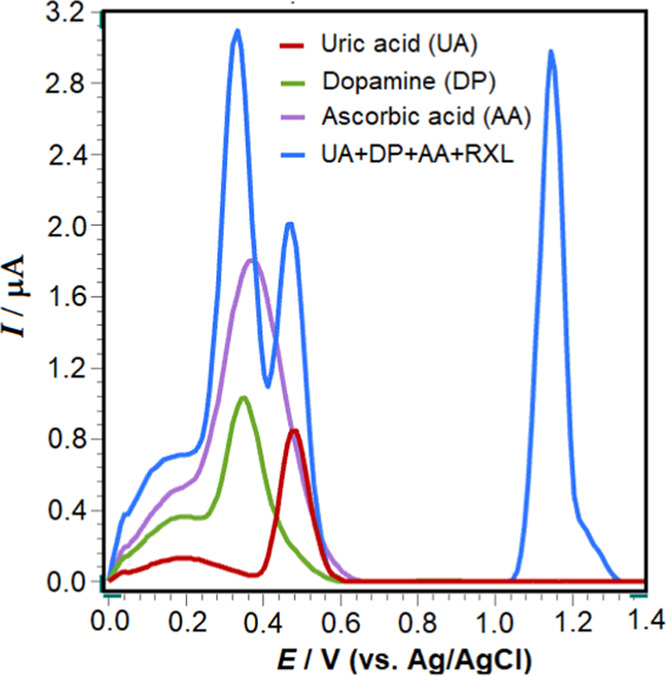
Square-wave voltammograms of 5 × 10^–7^ M RXL, 1 × 10^–6^ M UA, DP, and 5 × 10^–6^ M AA in pH 4.7 ABS. SWV parameters are as follows: Δ*E*_s_, 20 mV; *f*, 100 Hz; and Δ*E*_sw_, 40 mV. The voltammogram depicted by the blue line includes AA, UA, DP, and RXL.

To assess the selectivity of the proposed voltammetric method, substances potentially interfering with the 0.6 μM RXL solution were tested. No significant change in the peak potential of RXL was observed upon the addition of Ca^2+^, Cu^2+^, Zn^2+^, Ag^+^, Na^+^, NO_3_^–^, Cl^–^, uric acid (UA), ascorbic acid (AA), and dopamine (DP) compounds. The oxidation potential of RXL was observed at +1.15 V, while UA exhibited oxidation at approximately +0.48 V. DP and AA showed peak potentials at +0.35 and +0.37 V, respectively (Figure 10). These findings suggest that the method developed using the poly(taurine)-CP electrode is highly selective.

After the selectivity was tested, the practical applicability of the proposed procedure was investigated using commercially available tablet forms and model human urine samples. Detailed explanations of the sample preparation procedures outlined in Section 2.2.1. and 2.2.2. are provided in the following sections. Tablet solutions were easily prepared by diluting them to the target concentration within the working linear range of the final solution without the need for any sample extraction or filtration. The assay results with recoveries for the tested formulation are summarized in Table 2. These findings from the analysis of pharmaceutical products confirm that the proposed protocol was not significantly affected by any matrix effect.

**Table 2:** Analysis of Jakavi Tablets Containing RXL was Conducted by using the Proposed Method

labeled claim (mg)	10.0
Amound found^a^ (mg)	9.74 ± 0.86
%RSD	3.89
average recovery^a^ (%)	97.5 ± 0.75
RSD of recovery (%)	3.16

a Mean of five experiments.

**Figure 11:**
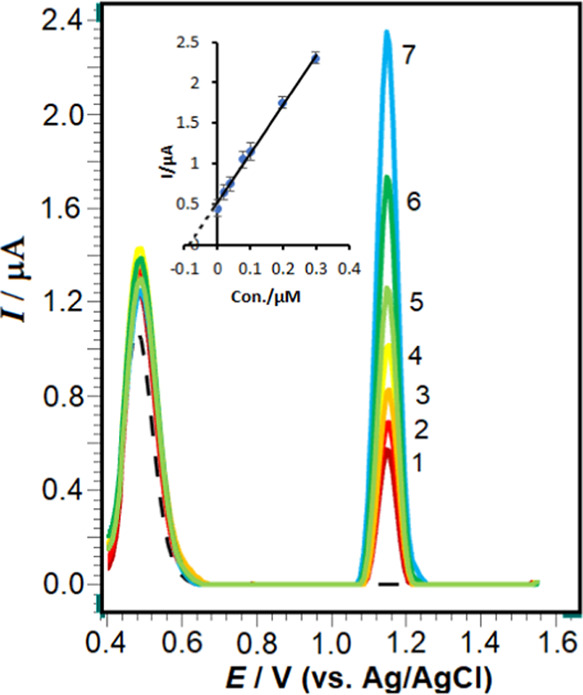
SWVs of the urine sample; dashed line depicts the diluted urine sample in the presence of 0.05 μM RXL (1), (2–7) after standard additions of 0.02, 0.04, 0.08, 0.1, 0.2, and 0.3 μM RXL in pH 4.7 ABS. Inset shows the result of the analysis by the standard addition method. SWV parameters are as follows: Δ*E*_s_, 14 mV; *f*, 100 Hz; and Δ*E*_sw_, 40 mV.

SW voltammetry signals for the analysis of urine samples are shown in Figure 11. The results are given in Table 3. As is known, the analysis of drugs taken from biological samples is quite time-consuming and requires the use of expensive organic solvents. With this technique, there is no pretreatment other than simply precipitating urine proteins with acetonitrile and diluting them with the chosen supporting electrolyte. Recovery results of RXL from urine solutions were calculated from the relevant linear regression equations given in Table 4. As can be seen in Figure 11, no substance and extra noise signals from the urine samples occurred in the potential range where the oxidation peak appeared.

**Table 3:** Regression Data of the Calibration Lines of RXL by SWV in a Human Urine Sample

parameters	oxidation of RXL
linearity range (μM)	0.02–0.3
slope (μA/μM)	6.0668
intercept (μA)	0.5158
correlation coefficient	0.998

**Table 4:** Results for Quantification and Recovery of RXL from the Urine Sample

	urine sample 1	urine sample 2	urine sample 3
added (μM)	0.04	0.04	0.04
found (μM)	0.039	0.042	0.041
recovery (%)^a^	97.5	105.0	102.5
bias (%)	2.5	–5.0	–2.5

a Mean of five experiments.

## 4 Conclusions

The preparation of taurine in an aqueous medium demonstrated excellent reproducibility and stability upon modification of the CP electrode using the CV electropolymerization technique. Results indicate that the poly(taurine)-CP electrode exhibits notable electrocatalytic activity toward the electrochemical oxidation of RXL, as evidenced by both CV and EIS analyses. Based on the findings, the electrode reaction of RXL on the poly(taurine)-CP electrode was evaluated as an irreversible and diffusion-controlled process. This work investigates the electroactivity of the JAK inhibitor RXL at the poly(taurine)-CP electrode. It elucidates that the compound undergoes irreversible oxidation at a potential of +1.15 V, indicative of an irreversible oxidation reaction across all pH values examined via cyclic voltammetric measurements. The linear working range was established as 1 × 10^–8^–1 × 10^–6^ M, with calculated LOD and LOQ values of 5 × 10^–9^ and 1 × 10^–8^ M, respectively. The method designed based on the poly(taurine)-CP electrode proved effective in determining RXL in real samples, such as tablet formulations and human urine samples. The obtained average recovery values were found to be 101.6 and 97.5%, respectively, when applied to spiked urine samples and tablet dosage forms. The excellent selectivity of the poly(taurine)-CP electrode enables accurate measurement of RXL in both human urine and tablet dosage forms, making this proposed polymer film electrode highly selective, easy to manufacture and use, cost-effective, reliable, and precise. It can be considered as a suitable alternative to other existing analytical methods.

## Supporting Information Available

Additional figures (S1–S5) as mentioned in the text (PDF)

## References

[ref1] Keenan C.; Nichols K. E.; Albeituni S. Use of the JAK inhibitor Ruxolitinib in the treatment of Hemophagocytic Lymphohistiocytosis. Front. Immunol. 2021, 12, 614704 10.3389/fimmu.2021.614704. 33664745 PMC7923355

[ref2] Gehringer M.; Forster M.; Laufer S. A. Solution-phase parallel synthesis of Ruxolitinib-derived Janus kinase inhibitors via copper-catalyzed Azide–Alkyne cycloaddition. ACS Comb. Sci. 2015, 17 (1), 5–10. 10.1021/co500122h. 25405713

[ref3] Hammersen J.; Birndt S.; Döhner K.; Reuken P.; Stallmach A.; Sauerbrey P.; La Rosée F.; Pfirrmann M.; Fabisch C.; Weiss M.; Träger K.; Bremer H.; Russo S.; Illerhaus G.; Drömann D.; Schneider S.; La Rosée P.; Hochhaus A. The JAK1/2 inhibitor ruxolitinib in patients with COVID-19 triggered hyperinflammation: the RuxCoFlam trial. Leukemia 2023, 37 (9), 1879–1886. 10.1038/s41375-023-01979-w. 37507425 PMC10457200

[ref4] Appeldoorn T. Y. J.; Munnink T. O.; Morsink L. M.; Hooge M. L. D.; Touw D. J. Pharmacokinetics and pharmacodynamics of ruxolitinib: a review. Clin. Pharmacokinet. 2023, 62 (4), 559–571. 10.1007/s40262-023-01225-7. 37000342 PMC10064968

[ref5] Botta C.; Indrieri A.; Garofalo E.; Biamonte F.; Bruni A.; Pasqua P.; Cesario F.; Costanzo F. S.; Longhini F.; Mendicino F. COVID-19: high-JAKing of the inflammatory “flight” by ruxolitinib to avoid the cytokine storm. Front. Oncol. 2021, 10, 599502 10.3389/fonc.2020.599502. 33489899 PMC7819896

[ref6] Damsky W.; King B. A. JAK inhibitors in dermatology: The promise of a new drug class. J. Am. Acad. Dermatol. 2017, 76, 736–744. 10.1016/j.jaad.2016.12.005. 28139263 PMC6035868

[ref7] Bose P.; Kuykendall A. T.; Miller C.; Kurtin S.; Farina K.; Harting D. M.; Mascarenhas J. O.; Mesa R. A. Moving beyond ruxolitinib failure in myelofibrosis: Evolving strategies for second line therapy. Expert Opin. Pharmacother. 2023, 24 (9), 1091–1100. 10.1080/14656566.2023.2213435. 37163478

[ref8] Chen W.; Jin W.; Fang H.; Chen H.; Chen X.; Zhang Y.; Hong Z. Development of a new taurine purity certified reference material. Microchem. J. 2022, 181, 107761 10.1016/j.microc.2022.107761.

[ref9] Daneshvar N.; Shirini F.; Langarudi M. S. N.; Karimi-Chayjani R. Taurine as a green bio-organic catalyst for the preparation of bio-active barbituric and thiobarbituric acid derivatives in water media. Bioorg. Chem. 2018, 77, 68–73. 10.1016/j.bioorg.2017.12.021. 29334621

[ref10] Manjunatha J. G.; Swamy B. K.; Deraman M.; Mamatha G. P. Simultaneous voltammetric measurement of ascorbic acid and dopamine at poly (vanillin) modified carbon paste electrode: A cyclic voltammetric study. Pharma. Chem. 2012, 4 (6), 2489–2497.

[ref11] Hasanzadeh M.; Pournaghi-Azar M. H.; Shadjou N.; Jouyban A. Electropolymerization of taurine on gold surface and its sensory application for determination of captopril in undiluted human serum. Mater. Sci. Eng., C 2014, 38, 197–205. 10.1016/j.msec.2014.01.051. 24656369

[ref12] Pandi K.; Sivakumar M.; Chen S. M.; Sakthivel M.; Raghavi G.; Chen T. W.; Liu Y. C.; Madhu R. Electrochemical synthesis of lutetium (III) hexacyanoferrate/poly (taurine) modified glassy carbon electrode for the sensitive detection of sulfite in tap water. J. Electrochem. Soc. 2018, 165, B469 10.1149/2.1171810jes.

[ref13] Madhu R.; Devadas B.; Chen S. M.; Rajkumar M. An enhanced direct electrochemistry of glucose oxidase at poly (taurine) modified glassy carbon electrode for glucose biosensor. Anal. Methods 2014, 6, 9053–9058. 10.1039/C4AY01406E.

[ref14] Wang Y.; Chen Z. A novel poly (taurine) modified glassy carbon electrode for the simultaneous determination of epinephrine and dopamine. Colloids Surf., B 2009, 74, 322–327. 10.1016/j.colsurfb.2009.07.046. 19716274

[ref15] Koçak Ç. C.; Altın A.; Aslışen B.; Koçak S. Electrochemical preparation and characterization of gold and platinum nanoparticles modified poly (taurine) film electrode and its application to hydrazine determination. Int. J. Electrochem. Sci. 2016, 11, 233–249. 10.1016/S1452-3981(23)15840-8.

[ref16] Wan Q.; Wang X.; Yu F.; Wang X.; Yang N. Poly(taurine)/MWNT-modified glassy carbon electrodes for the detection of acetaminophen. J. Appl. Electrochem. 2009, 39, 785–790. 10.1007/s10800-008-9721-1.

[ref17] Koçak Ç. C. Poly (taurine-glutathione)/carbon nanotube modified glassy carbon electrode as a new levofloxacin sensor. Electroanalysis 2019, 31, 1535–1544. 10.1002/elan.201900096.

[ref18] Thivya P.; Ramya R.; Wilson J. Poly (3, 4-ethylenedioxythiophene)/taurine biocomposite on screen printed electrode: Non-enzymatic cholesterol biosensor. Microchem. J. 2020, 157, 105037 10.1016/j.microc.2020.105037.

[ref19] Rajkumar M.; Li Y. S.; Chen S. M. Electrochemical detection of toxic ractopamine and salbutamol in pig meat and human urine samples by using poly taurine/zirconia nanoparticles modified electrodes. Colloids Surf., B 2013, 110, 242–247. 10.1016/j.colsurfb.2013.03.038. 23732800

[ref20] Rajkumar M.; Chiou S. C.; Chen S. M.; Thiagarajan A novel poly (taurine)/nano gold modified electrode for the determination of arsenic in various water samples. Int. J. Electrochem. Sci. 2011, 6, 3789–3800. 10.1016/S1452-3981(23)18289-7.

[ref21] Di Michele A.; Schoubben A.; Varfaj I.; D’Arpino A.; Mercolini L.; Sardella R.; Ricci M.; Tiacci E. Improved achiral and chiral HPLC-UV analysis of ruxolitinib in two different drug formulations. Separations 2020, 7, 47 10.3390/separations7030047.

[ref22] Charlier B.; Marino L.; Dal Piaz F.; Pingeon M.; Coglianese A.; Izzo B.; Serio B.; Selleri C.; Filippelli A.; Izzo V. Development and validation of a reverse-phase high-performance liquid chromatography with fluorescence detection (RP-HPLC-FL) method to quantify ruxolitinib in plasma samples. Anal. Lett. 2019, 52, 1328–1339. 10.1080/00032719.2018.1537283.

[ref23] Satyanarayana P. V. V.; Madhavi A. S. A novel RP-HPLC method for the quantification of ruxolitinib in formulations. J. Atoms Mol. 2012, 2, 223.

[ref24] Biswal S.; Mondal S.; Mondal P. A New Stability Indicating High Performance Liquid Chromatography Method for the Estimation of Ruxolitinib in Bulk and Tablet Dosage Form. Pharm. Methods 2019, 10 (2), 53–57. 10.5530/phm.2019.2.10.

[ref25] Tachet J.; Versace F.; Mercier T.; Buclin T.; Decosterd L. A.; Choong E.; Girardin F. R. Development and validation of a multiplex HPLC-MS/MS assay for the monitoring of JAK inhibitors in patient plasma. J. Chromatogr. B: Anal. Technol. Biomed. Life Sci. 2023, 1230, 123917 10.1016/j.jchromb.2023.123917. 37956468

[ref26] Al-Hossaini A. M.; Al-Mutairi A. S.; Darwish I. A.; Bacheit A. H.; Ali A. M.; Darwish H. W. Novel microwell-based spectrofluorimetric method assisted with fluorescence plate reader for determination of ruxolitinib: Optimization by response surface methodology and application to in-vitro drug release, content uniformity testing and analysis of u···. Talanta Open 2023, 7, 100207 10.1016/j.talo.2023.100207.

[ref27] Tallam A. K.; Alapati S.; Nuli M. V. A review on bioanalytical method development and validation of anticancer drugs by using lc/ms/ms and its applications on routine analysis. J. Integral Sci. 2023, 4–19. 10.37022/jis.v6i1.51.

[ref28] Aydin I.; Akgun H.; Talay Pınar P. Analytical determination of the oxazolidinone antibiotic linezolid at a pencil graphite and carbon paste electrodes. ChemistrySelect 2019, 4, 9966–9971. 10.1002/slct.201902269.

[ref29] Talay Pınar P.; Yardım Y.; Gülcan M.; Şentürk Z. The first approach for the simultaneous quantification of isoproturon, carbendazim, and carbofuran at the surface of a MIL-101 (Cr) metal-organic framework-based electrode. Inorg. Chem. Commun. 2023, 111327.

[ref30] Ali H. S.; Barzani H. A. H.; Yardım Y. Utilizing epicatechin voltammetric oxidation signal for the estimation of total phenolic content in the tea samples via the unmodified boron-doped diamond electrode surface. Microchem. J. 2023, 189, 108572 10.1016/j.microc.2023.108572.

[ref31] Mohammed M. A.; Attia A. K.; Elwy H. M. Electrochemical sensor based on multiwalled carbon nanotube, alizarine red S and chitosan for simultaneous determination of oxomemazine hydrochloride, paracetamol and guaifenesin. Electroanalysis 2017, 29 (2), 506–513. 10.1002/elan.201600311.

[ref32] El-Gendy D. M.; Mohamed M. A.; Amirghasemi F.; Atty S. A.; Abd El-Rahman M. K.; Mousavi M. P. From aluminum foil to personalized medicine: Ecofriendly one-step electrode modification for rapid detection of ertapenem and co-administered medications. J. Sci.: Adv. Mater. Devices 2023, 8 (3), 100601 10.1016/j.jsamd.2023.100601.

[ref33] Khazaee Nejad S.; Ma H.; Al-Shami A.; Soleimani A.; Mohamed M. A.; Dankwah P.; Lee H. J.; Mousavi M. P. Sustainable agriculture with LEAFS: a low-cost electrochemical analyzer of foliage stress. Sens. Diagn. 2024, 3, 400–411. 10.1039/D3SD00296A. PMC12064159 40352404

[ref34] Mohamed M. A.; Eldin G. M.; Ismail S. M.; Zine N.; Elaissari A.; Jaffrezic-Renault N.; Errachid A. Innovative electrochemical sensor for the precise determination of the new antiviral COVID-19 treatment Favipiravir in the presence of coadministered drugs. J. Electroanal. Chem. 2021, 895, 115422 10.1016/j.jelechem.2021.115422. PMC8161794 34075313

[ref35] Manjunatha J. G.; Swamy B. K.; Deepa R.; Krishna V.; Mamatha G. P.; Chandra U.; Shankar S. S.; Sherigara B. S. Electrochemical studies of dopamine at eperisone and cetyl trimethyl ammonium bromide surfactant modified carbon paste electrode: a cyclic voltammetric study. Int. J. Electrochem. Sci. 2009, 4 (5), 662–671. 10.1016/S1452-3981(23)15172-8.

[ref36] Darabi R.; Shabani-Nooshabadi M.; Khoobi A. A potential strategy for simultaneous determination of deferoxamine and vitamin C using MCR-ALS with nanostructured electrochemical sensor in serum and urine of thalassemia and diabetic patients. J. Electrochem. Soci. 2021, 168 (4), 046514 10.1149/1945-7111/abf6ed.

[ref37] Tigari G.; Manjunatha J. G. Optimized voltammetric experiment for the determination of phloroglucinol at surfactant modified carbon nanotube paste electrode. Instrum. Exp. Tech. 2020, 63, 750–757. 10.1134/S0020441220050139.

[ref38] Ghoreishi S. M.; Behpour M.; Khoobi A.; Masoum S. Application of experimental design for quantification and voltammetric studies of sulfapyridine based on a nanostructure electrochemical sensor. Arab. J. Chem. 2017, 10, S3156–S3166. 10.1016/j.arabjc.2013.12.009.

[ref39] Khoobi A.; Attaran A. M.; Yousofi M.; Enhessari M. A sensitive lead titanate nano-structured sensor for electrochemical determination of pentoxifylline drug in real samples. J. Nanostruct. Chem. 2019, 9, 29–37. 10.1007/s40097-019-0295-8.

[ref40] Mollaei M.; Ghoreishi S. M.; Khoobi A. Electrochemical investigation of a novel surfactant for sensitive detection of folic acid in pharmaceutical and biological samples by multivariate optimization. Measurement 2019, 145, 300–310. 10.1016/j.measurement.2019.05.064.

[ref41] Manjunatha J. G.; Swamy B. K.; Mamatha G. P.; Gilbert O.; Srinivas M. T.; Sherigara B. S. Electrochemical studies of clozapine drug using carbon nanotube-SDS modified carbon paste electrode: A cyclic voltammetry study. Der. Pharma Chemica 2011, 3 (2), 236–249.

[ref42] Manjunatha J. G.; Swamy B. K.; Mamatha G. P.; Gilbert O.; Chandrashekar B. N.; Sherigara B. S. Electrochemical studies of dopamine and epinephrine at a poly (tannic acid) modified carbon paste electrode: a cyclic voltammetric study. Int. J. Electrochem. Sci. 2010, 5 (9), 1236–1245. 10.1016/S1452-3981(23)15358-2.

[ref43] Pushpanjali P. A.; Manjunatha J. G.; Hareesha N.; D’Souza E. S.; Charithra M. M.; Prinith N. S. Voltammetric analysis of antihistamine drug cetirizine and paracetamol at poly (L-Leucine) layered carbon nanotube paste electrode. Surf. Interfaces 2021, 24, 101154 10.1016/j.surfin.2021.101154.

[ref44] Hareesha N.; Manjunatha J. G. Electro-oxidation of formoterol fumarate on the surface of novel poly (thiazole yellow-G) layered multi-walled carbon nanotube paste electrode. Sci. Rep. 2021, 11 (1), 12797 10.1038/s41598-021-92099-x. 34140565 PMC8211837

[ref45] Manjunatha J. G.; Swamy B. K.; Gilbert O.; Mamatha G. P.; Sherigara B. S. Sensitive voltammetric determination of dopamine at salicylic acid and TX-100, SDS, CTAB modified carbon paste electrode. Int. J. Electrochem. Sci. 2010, 5 (5), 682–695. 10.1016/S1452-3981(23)15315-6.

[ref46] Aydın E. B.; Aydın M.; Sezgintürk M. K. A simple and low-cost electrochemical immunosensor for ultrasensitive determination of calreticulin biomarker in human serum. Macromol. Biosci. 2023, 23, 2200390 10.1002/mabi.202200390. 36419333

[ref47] Khoobi A.; Ghoreishi S. M.; Masoum S.; Behpour M. Multivariate curve resolution-alternating least squares assisted by voltammetry for simultaneous determination of betaxolol and atenolol using carbon nanotube paste electrode. Bioelectrochemistry 2013, 94, 100–107. 10.1016/j.bioelechem.2013.04.002. 23632433

[ref48] Ghoreishi S. M.; Behpour M.; Khoobi A.; Salavati-Niasari M. Electrochemical study of a self-assembled monolayer of N, N′-bis [(E)-(1-pyridyl) methylidene]-1, 3-propanediamine formed on glassy carbon electrode: preparation, characterization and application. Anal. Methods 2013, 5 (23), 6727–6733. 10.1039/c3ay41480a.

[ref49] Kassa A.; Bitew Z.; Abebe A. A non-toxic poly (resorcinol) modified glassy carbon electrode for highly selective square wave voltammetry determination of aspirin in tablet formulations and human urine samples. Sens. Bio-Sensing Res. 2023, 39, 100554 10.1016/j.sbsr.2023.100554.

[ref50] Vijayarohini P.; Mercy A. S. C.; Kavitha G.; Alwar S. B. S. Enhanced biological properties of taurine metal complexes via binding active sites. Mater. Today: Proc. 2020, 33, 2631–2640. 10.1016/j.matpr.2020.01.217.

[ref51] Ren G.; Li B.; Ren L.; Lu D.; Zhang P.; Tian L.; Di W.; Shao W.; He J.; Sun D. pH-responsive nanoemulsions based on a dynamic covalent surfactant. Nanomaterials 2021, 11 (6), 1390 10.3390/nano11061390. 34070322 PMC8227844

[ref52] Wu C. P.; Hsu S. C. Small Molecule Chemosensitizing Agents: Polo-Like Kinase 1 (Plk1), BRAF and Janus Kinase (JAK) Inhibitors. In Protein Kinase Inhibitors as Sensitizing Agents for Chemotherapy; Academic Press, 2019; pp 169–185.

[ref53] Çorman M. E.; Cetinkaya A.; Ozcelikay G.; Özgür E.; Atici E. B.; Uzun L.; Ozkan S. A. A porous molecularly imprinted nanofilm for selective and sensitive sensing of an anticancer drug ruxolitinib. Anal. Chim. Acta 2021, 1187, 339143 10.1016/j.aca.2021.339143. 34753569

[ref54] Bilge S.; Karadurmus L.; Atici E. B.; Sınağ A.; Ozkan S. A. Electrochemical investigation of ruxolitinib: Sensitive voltammetric assay in drug product and human serum by using different solid electrodes. Electroanalysis 2022, 34, 1318–1328. 10.1002/elan.202100625.

[ref55] Bilge S.; Karadurmus L.; Atici E. B.; Sınağ A.; Ozkan S. A. A novel electrochemical sensor based on magnetic Co3O4 nanoparticles/carbon recycled from waste sponges for sensitive determination of anticancer drug ruxolitinib. Sens. Actuators, B 2022, 367, 132127 10.1016/j.snb.2022.132127.

[ref56] Tsai T. H.; Chen T. W.; Chen S. M.; Sarawathi R. Nickel, copper and manganese hexacyanoferrate with poly (3, 4-ethylenedioxythiophene) hybrid film modified electrode for selectively determination of ascorbic acid. Russ. J. Electrochem. 2012, 48, 291–301. 10.1134/S1023193512030147.

