# Supplementary material for: Electrochemical Behavior of Janus Kinase Inhibitor Ruxolitinib at a Taurine-Electropolymerized Carbon Paste Electrode: Insights into Sensing Mechanisms
Source: ACS Appl Bio Mater. 2024 Apr 6;7(5):3179–89. doi: 10.1021/acsabm.4c00186 (PMC11110052; doi:10.1021/acsabm.4c00186)
Supplement: Supplementary file 1 — mt4c00186_si_001.pdf [file mt4c00186_si_001.pdf]

Supporting Information

Electrochemical Behavior of Janus kinase inhibitor Ruxolitinib at a Taurine Electropolymerized Carbon Paste Electrode: Insights into Sensing Mechanisms

Hasret Subak, Pınar Talay Pınar*

Department of Analytical Chemistry, Faculty of Pharmacy, Van Yuzuncü Yil University, Zeve Campus 65080 Van, Turkey

*Corresponding Authors; e-mail: ptalay@gmail.com (P. Talay Pınar) Fax: 90 432 225 18 06 Website: https://avesis.yyu.edu.tr/ptalay

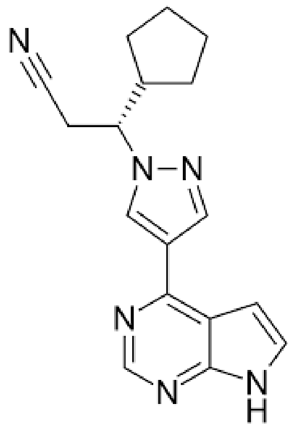

Figure S1. Chemical structures of Ruxolitinib

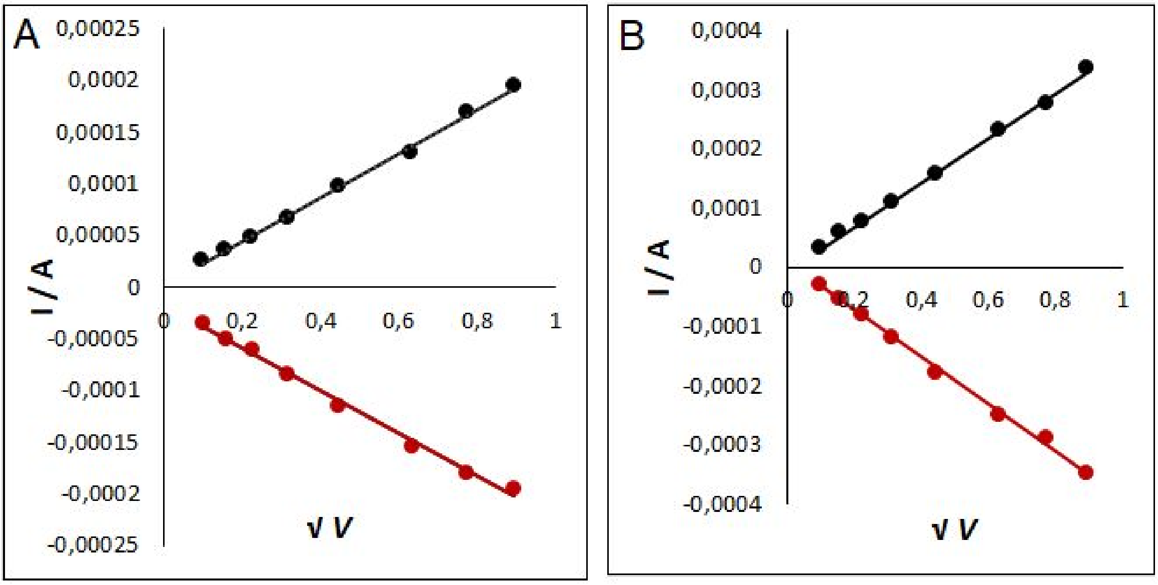

Figure S2. The graphs of 2.0 mM $[Fe(CN)_6]^{3-/4-}$ in 0.5 M KCl obtained at (A) CPE, and (B) poly(Taurine)/CPE at different scan rates (0.01–0.8 V/s). Figure insets indicate relation between the anodic peak current (**black**) and or the cathodic peak current (**red**) and square root of the scan rate.

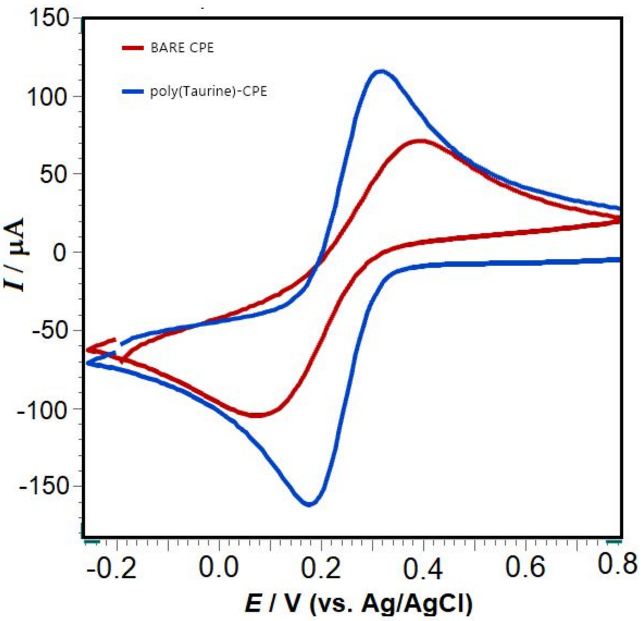

Figure S3. CV voltammograms of 2.0 mM $[Fe(CN)_6]^{3-/4-}$ in 0.5 M KCl at CP (red) and poly(Taurine)-CP (blue) electrodes at 100 mV/s.

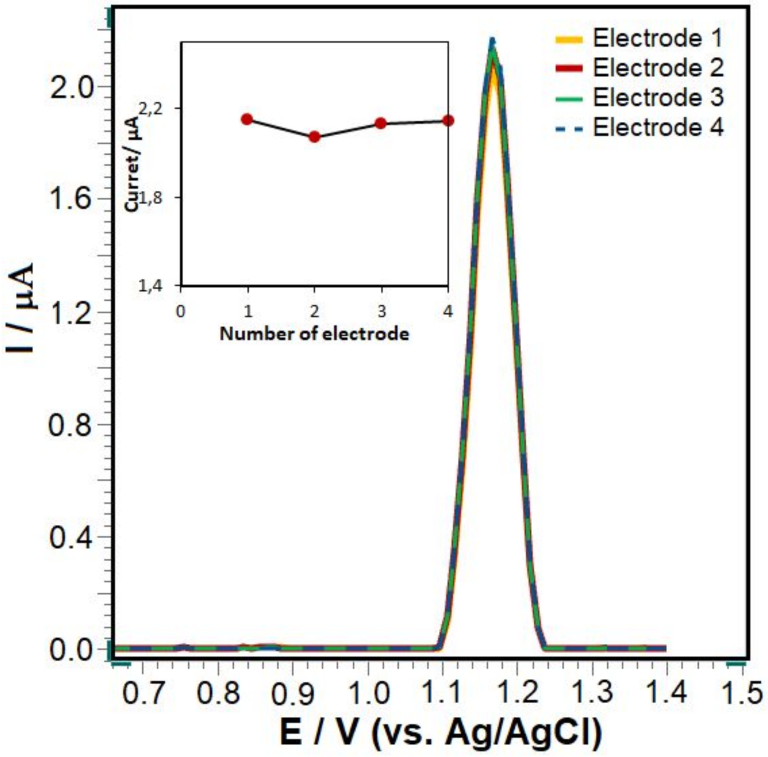

Figure S4. Plot of peak current responses of four different poly (Taurine)/ CP electrode.

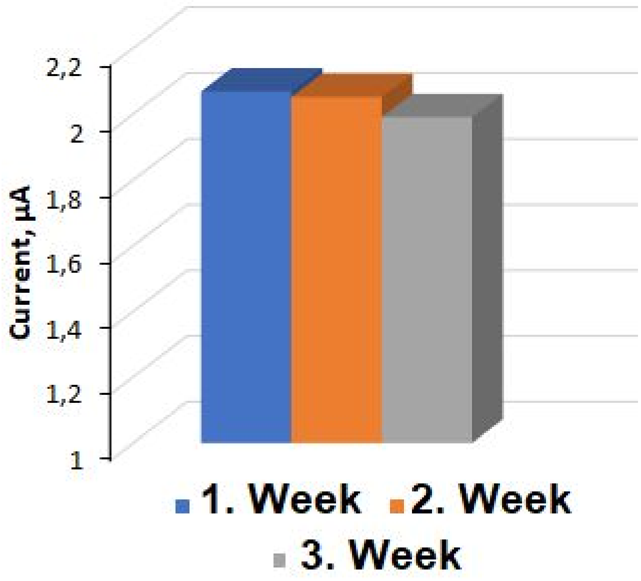

Figure S5. Current response of poly (Taurine)/ CP electrode to RXL over a period of three weeks
